# Supplementary material for: No evidence for increased transmissibility from recurrent mutations in SARS-CoV-2
Source: Nat Commun. 2020 Nov 25;11:5986. doi: 10.1038/s41467-020-19818-2 (PMC7688939; doi:10.1038/s41467-020-19818-2)
Supplement: Supplementary file 1 — Supplementary Information [file 41467_2020_19818_MOESM1_ESM.pdf]

Supplementary Material

No evidence for increased transmissibility from recurrent mutations in SARS-CoV-2

Lucy van Dorp[1*+]
Damien Richard[2,3*]
Cedric CS. Tan[1]
Liam P. Shaw[4]
Mislav Acman[1]
François Balloux[1+]

[1] UCL Genetics Institute, University College London, London WC1E 6BT, UK
[2] Cirad, UMR PVBMT, F-97410 St Pierre, Réunion, France
[3] Université de la Réunion, UMR PVBMT, F-97490 St Denis, Réunion, France
[4] Nuffield Department of Medicine, John Radcliffe Hospital, University of Oxford, Oxford OX3 9DU, UK

*contributed equally
+ corresponding; lucy.dorp.12@ucl.ac.uk (Lucy van Dorp) and f.balloux@ucl.ac.uk (François Balloux)

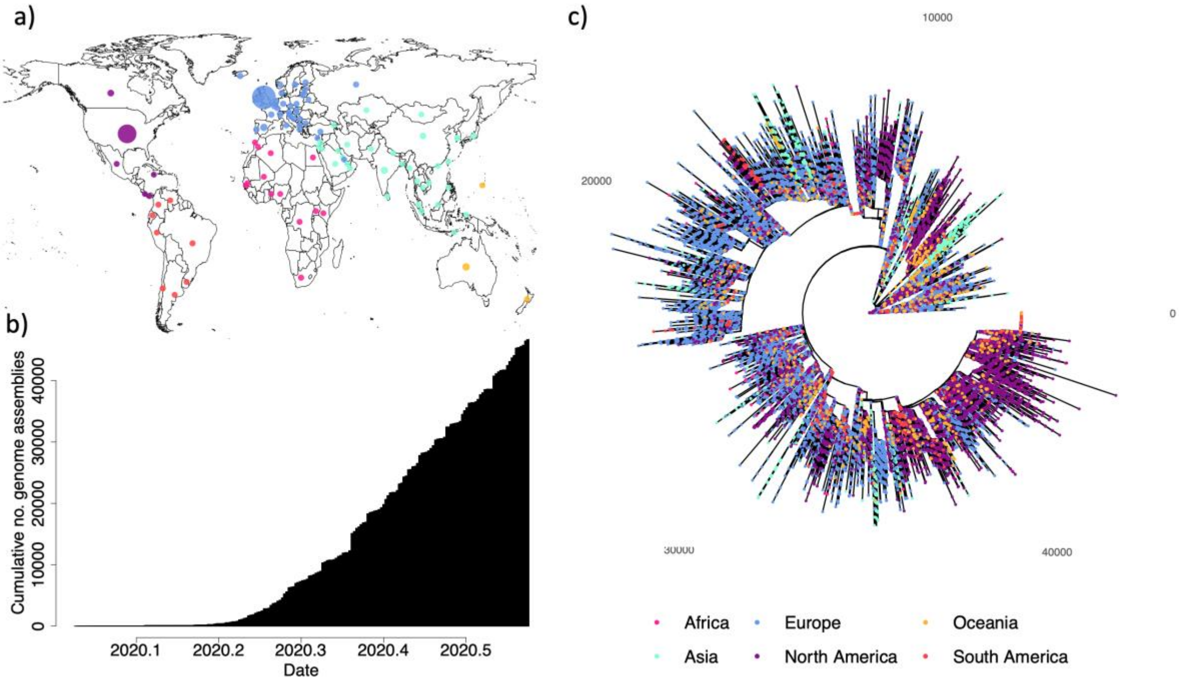

Figure S1: a) Geographic origin of sequences included in phylogenetic analysis (downloaded 30/07/2020). Purple: North America, coral: South America, blue: Europe, pink: Africa, green: Asia, Oceania: orange. Circles are proportional to the number of uploaded assemblies. b) Date of sample collection of all assemblies included in the dataset. c) Maximum likelihood phylogenetic tree (radial format). Tips are annotated by location of sampling as given in panel a). The map in Figure 1B was created using the R package rworldmap using the public domain Natural Earth data set.

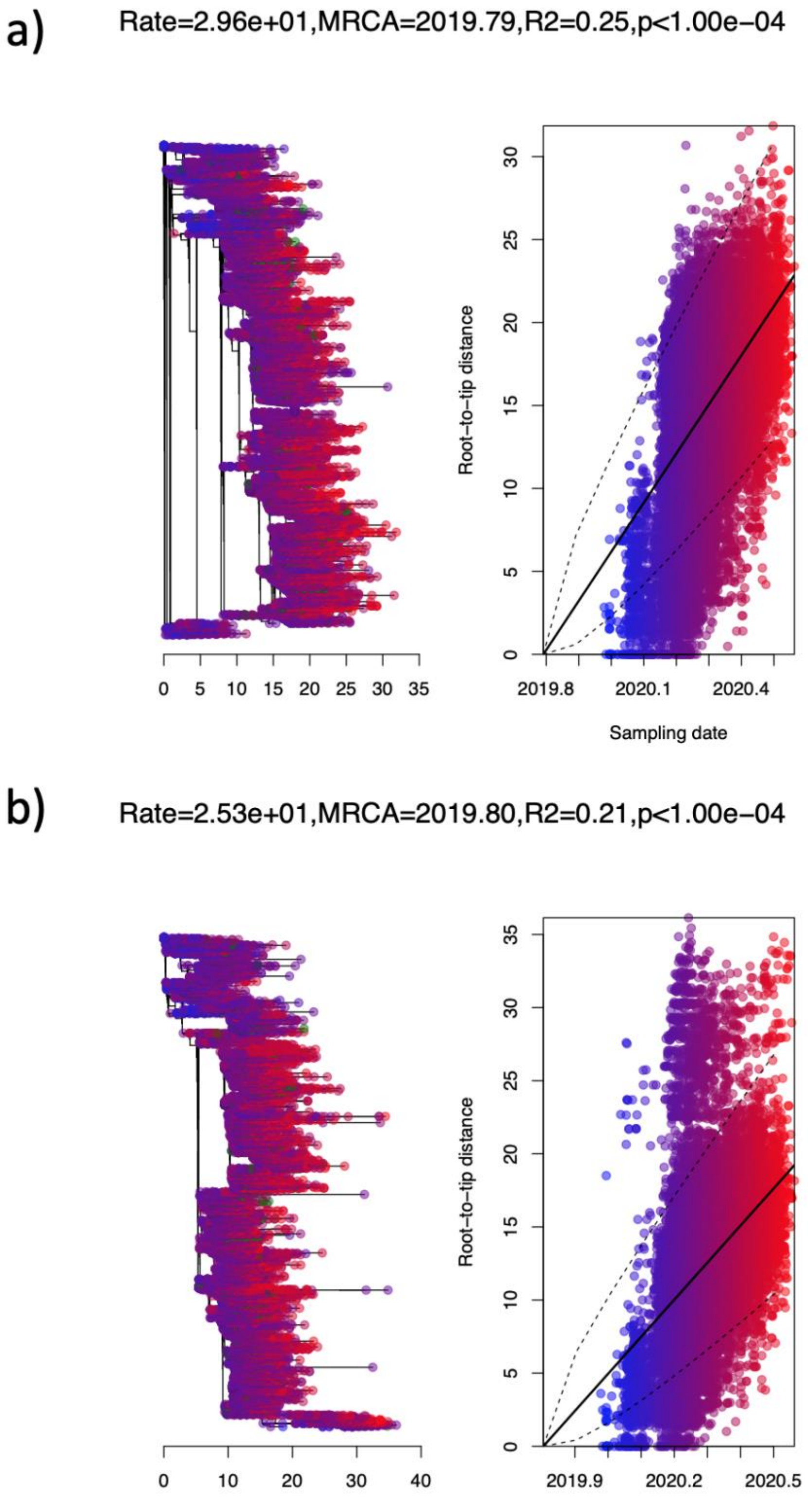

Figure S2: Root-to-tip regression performed using the *roottotip()* function provided by BactDating (see main text Methods) for both masked alignments (a- de Maio *et al.*, b – NextStrain). X-axis provides the time of sampling with y providing the root-to-tip distance across the rooted maximum likelihood phylogenetic tree. BactDating provides the empirical *p*-value following 10,000 permutations of the tree sampling dates. In both cases *p*<1e-04.

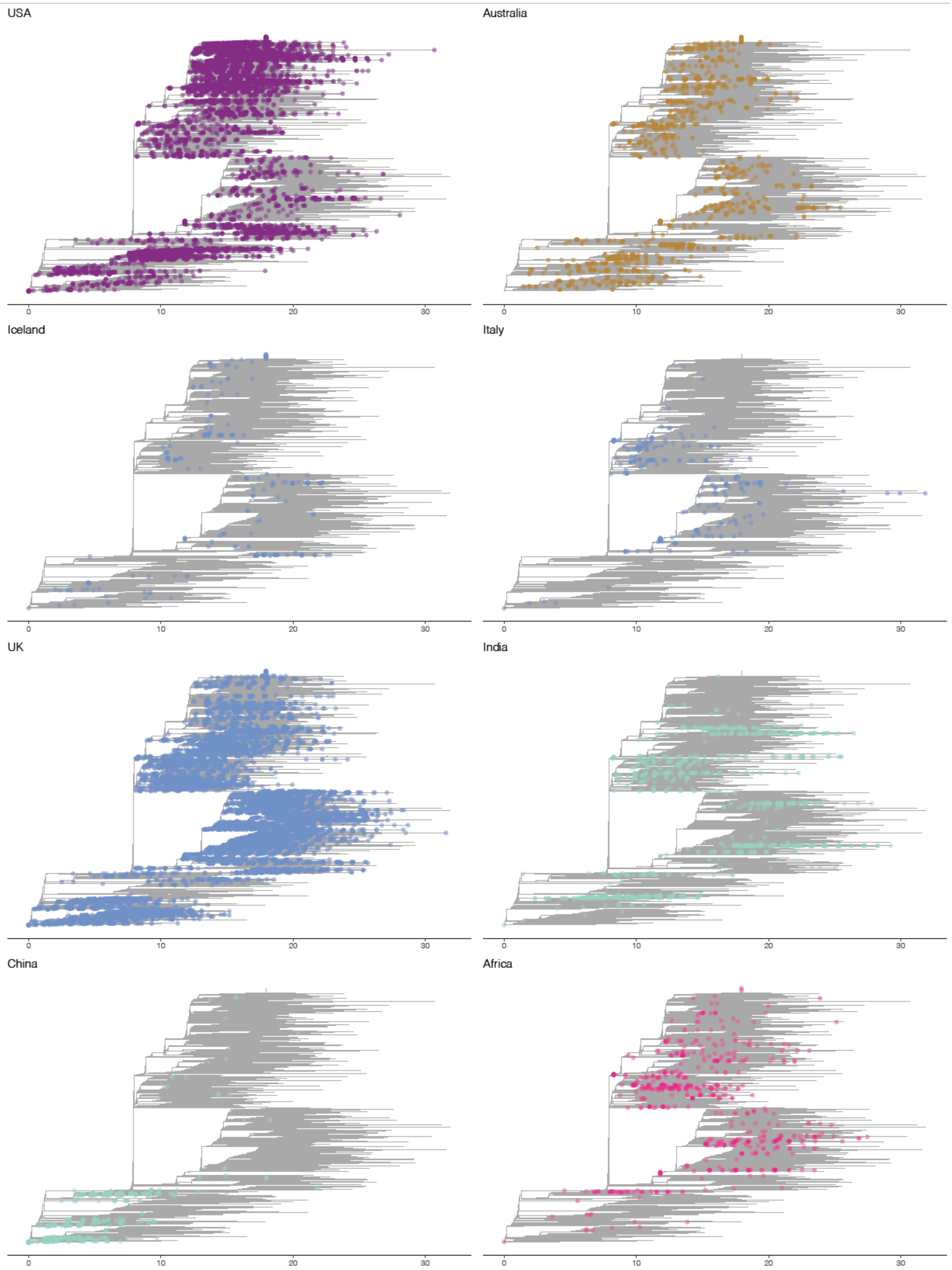

Figure S3: Maximum likelihood phylogenetic trees rooted on Wuhan-Hu-1 built using the Augur tree rapid phylodynamic pipeline. Tips are coloured by regions as given in main text Figure 1 and Figure S1. Genomes sampled in specific geographic regions are highlighted as indicated on the top-left of each plot.

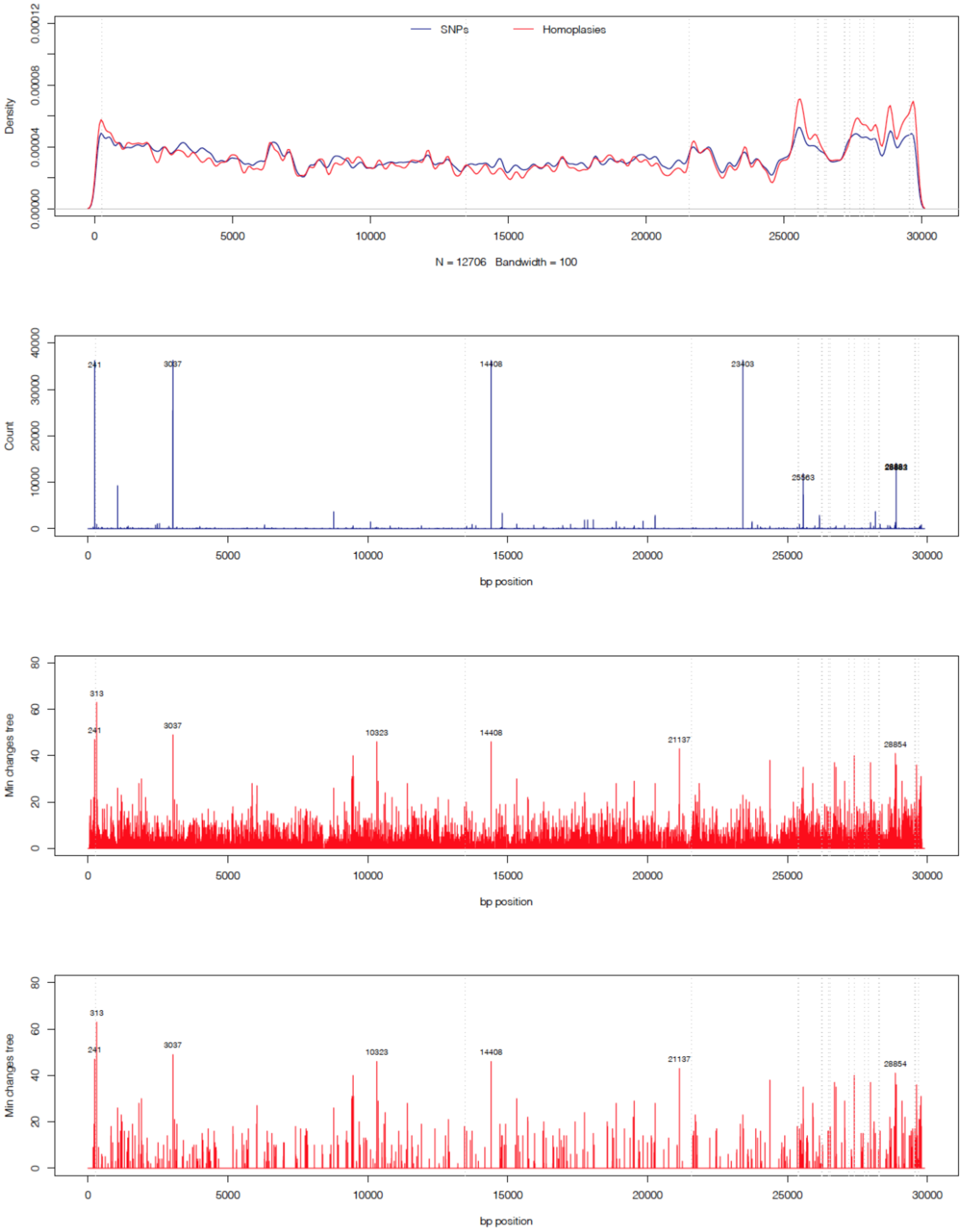

Figure S4: Genome-wide distribution of SNPs and homoplasies in SARS-CoV-2. Top provides the density of raw inferred SNPs and homoplasies genome-wide. The SNP count is provided with SNPs occurring in >10,000 isolates annotated. The raw count of 5,710 homoplasies is given in red with those responsible for >40 minimum changes on the tree annotated. This is filtered to a final set of 398 recurrent mutations (bottom panel), again annotated for those contributing to >40 minimum changes on the tree. A full list of filtered and non-filtered homoplasies for this alignment is provided in **Data 3.**

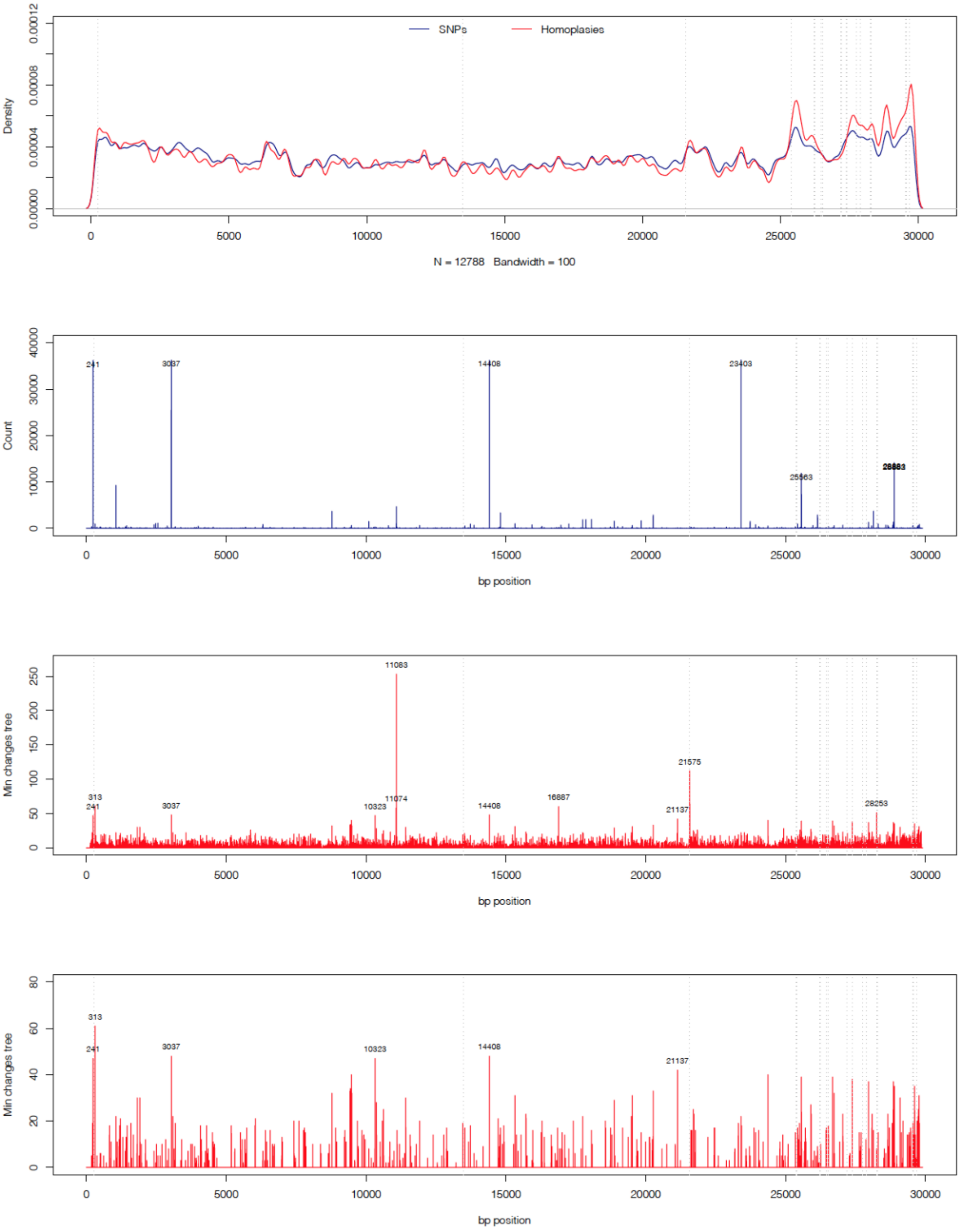

Figure S5: Genome-wide distribution of SNPs and homoplasies in SARS-CoV-2 following the NextStrain masking strategy (see **Data 5**). Top provides the density of raw inferred SNPs and homoplasies genome-wide. The SNP count is provided with SNPs occurring in >10,000 isolates annotated. The raw count of 5,793 homoplasies is given in red with those responsible for >40 minimum changes on the tree annotated. This is filtered to a final set of 411 recurrent mutations (bottom panel), again annotated for those contributing to >40 minimum changes on the tree. A full list of filtered and non-filtered homoplasies for this alignment is provided in **Data 3.**

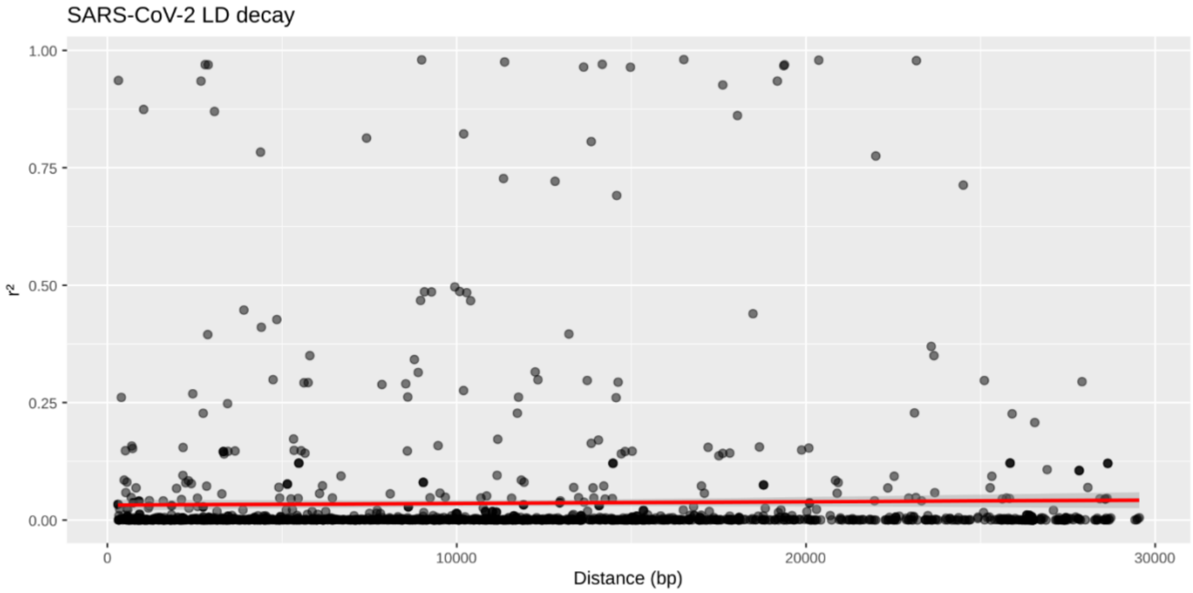

Figure S6 Linkage disequilibrium decay patterns in SARS-CoV-2. The y-axis provides the linkage disequilibrium (r^2) between all pairwise combinations of variant sites, with the x-axis providing the distance between these sites in base pair units. The linear regression of the relationship between linkage disequilibrium and distance between the SNPs yielded a regression coefficient of $3.56e^{-7}$ and a proportion of explained variance (R squared) of $4.52e^{-4}$.

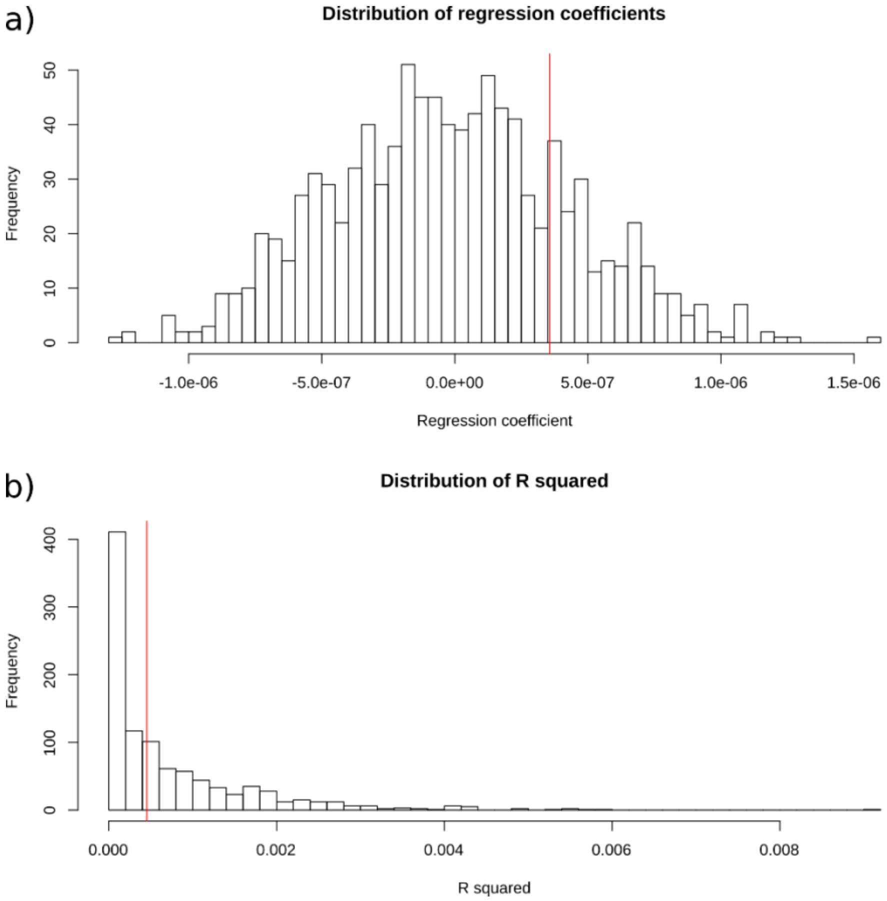

Figure S7: a) fitted regression coefficient and b) R^2 (red lines) compared to 1,000 permutations of genome coordinates. In both cases the values obtained for the SARS-CoV-2 alignment fall within the null distribution: 44.7% of randomized r^2 values are greater than the observed r^2 value and 21.0% of the randomized regression coefficients are greater than the observed value.

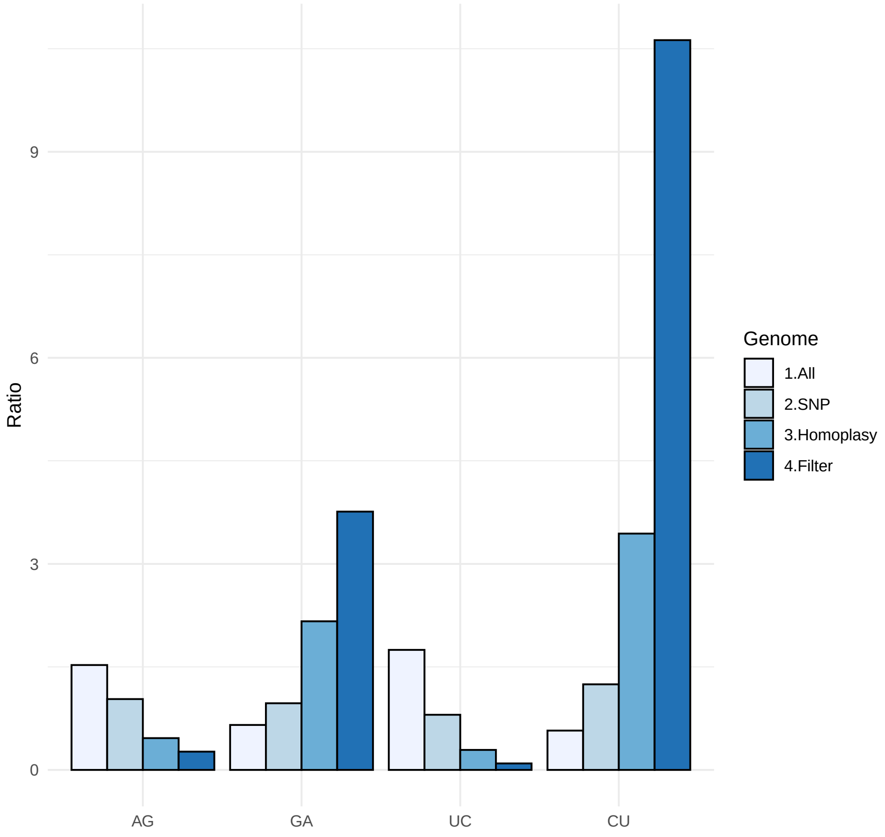

Figure S8: Ratio of observed bases (AG:A/G, GA:G/A, UC:T/C, CU:C.T) genome-wide, for solely SNP sites, for homoplasic positions and for filtered homoplasies.

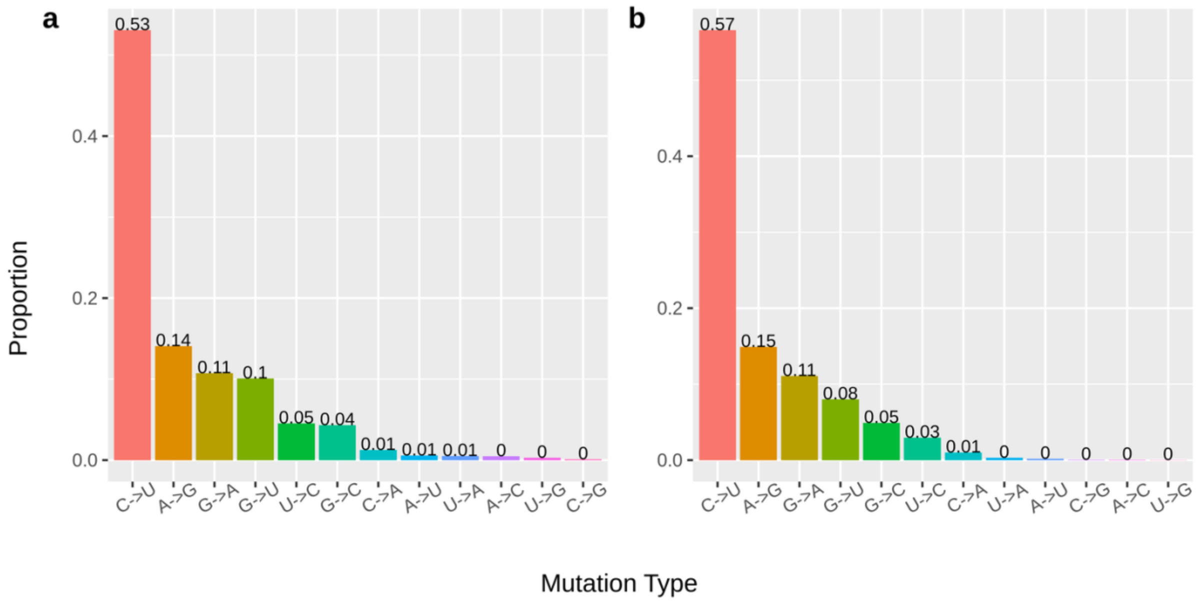

Figure S9. Bar plots of the cumulative frequencies of each type of SNP (A) across the entire SARS-CoV-2 genome and (B) across the 308 filtered homoplasic sites. Proportions were computed by dividing frequency of each SNP by the total number of SNPs across all genomes.

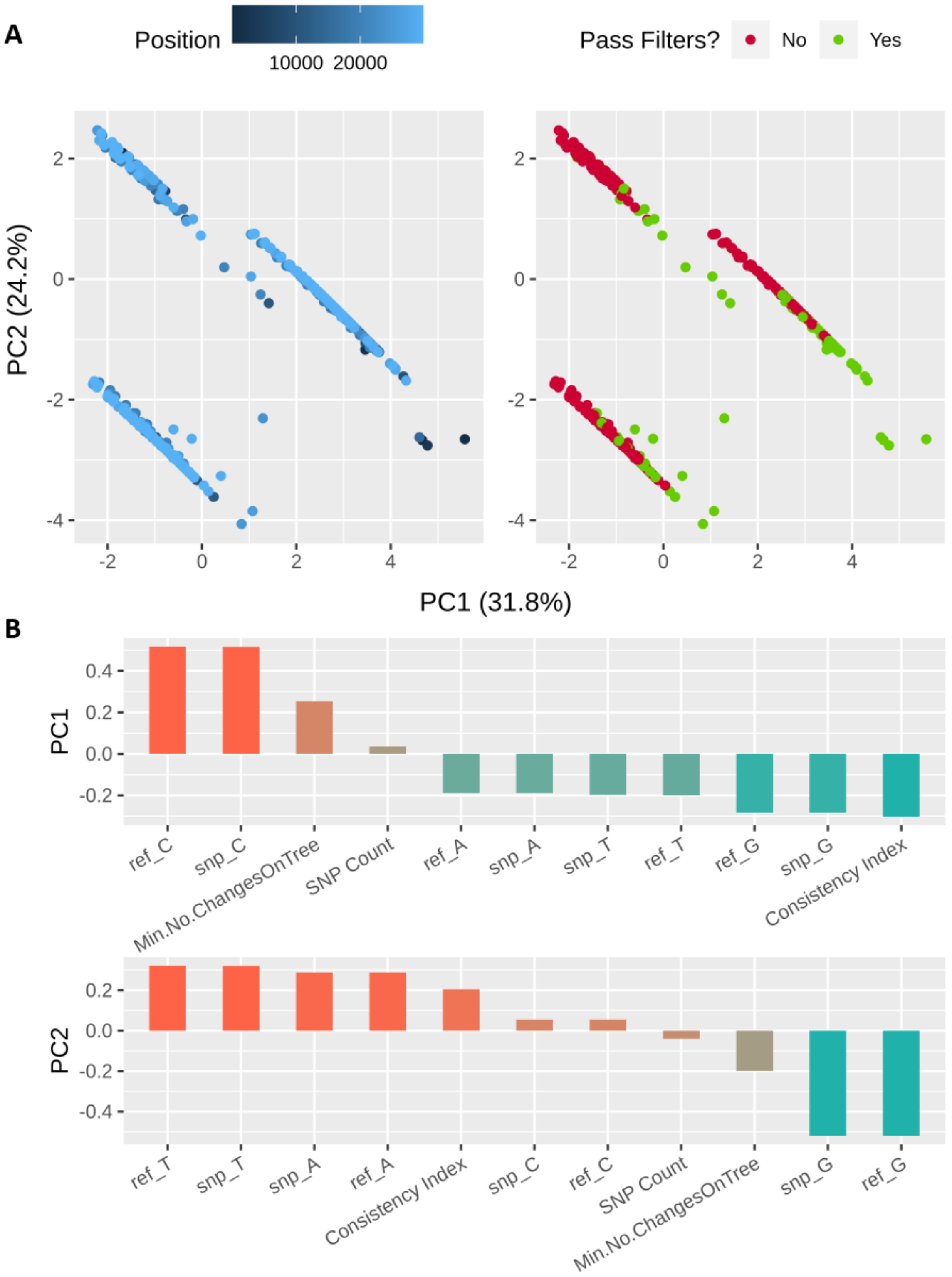

Figure S10. (A) Projections of principle components 1 and 2, coloured by genome position on the Wuhan-Hu-1 reference, or by whether the homoplasies were retained after filtering. (B) Loadings of each input variable for principle components 1 and 2.

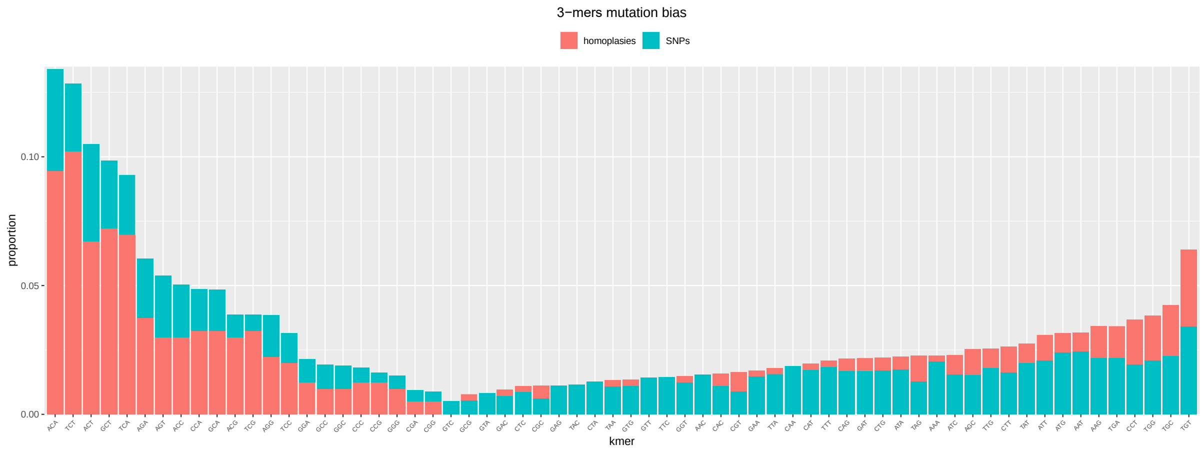

Figure S11: Proportion of 3-mers in referent SARS-CoV-2 genome containing a variable base in their central position. The proportions are coloured by filtered homoplastic status and given as the frequency relative to other sites in that state.

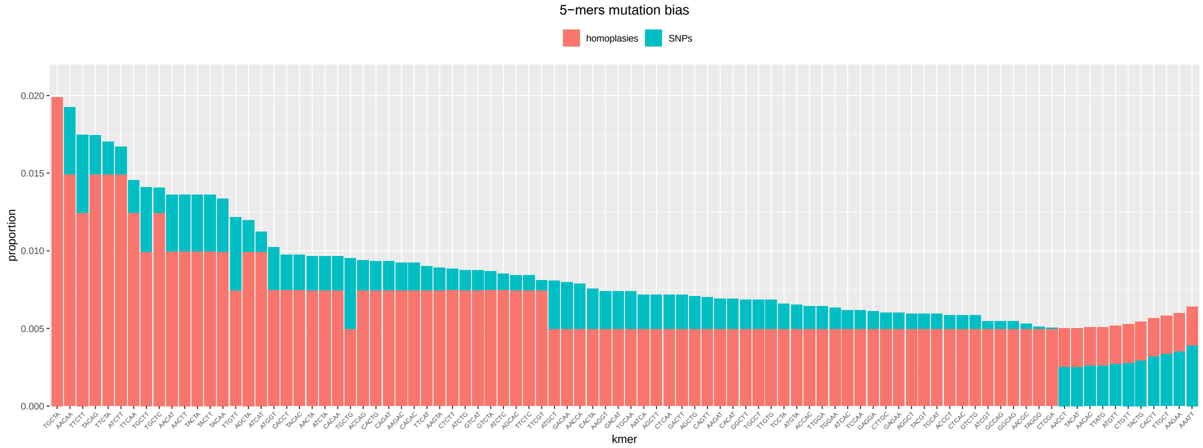

Figure S12: Proportion of 5-mers in referent SARS-CoV-2 genome containing a variable base in their central position. The proportions are coloured by the filtered homoplastic status and given as the frequency relative to other sites in that state. Sites with frequency lower than 0.005 were excluded from the figure.

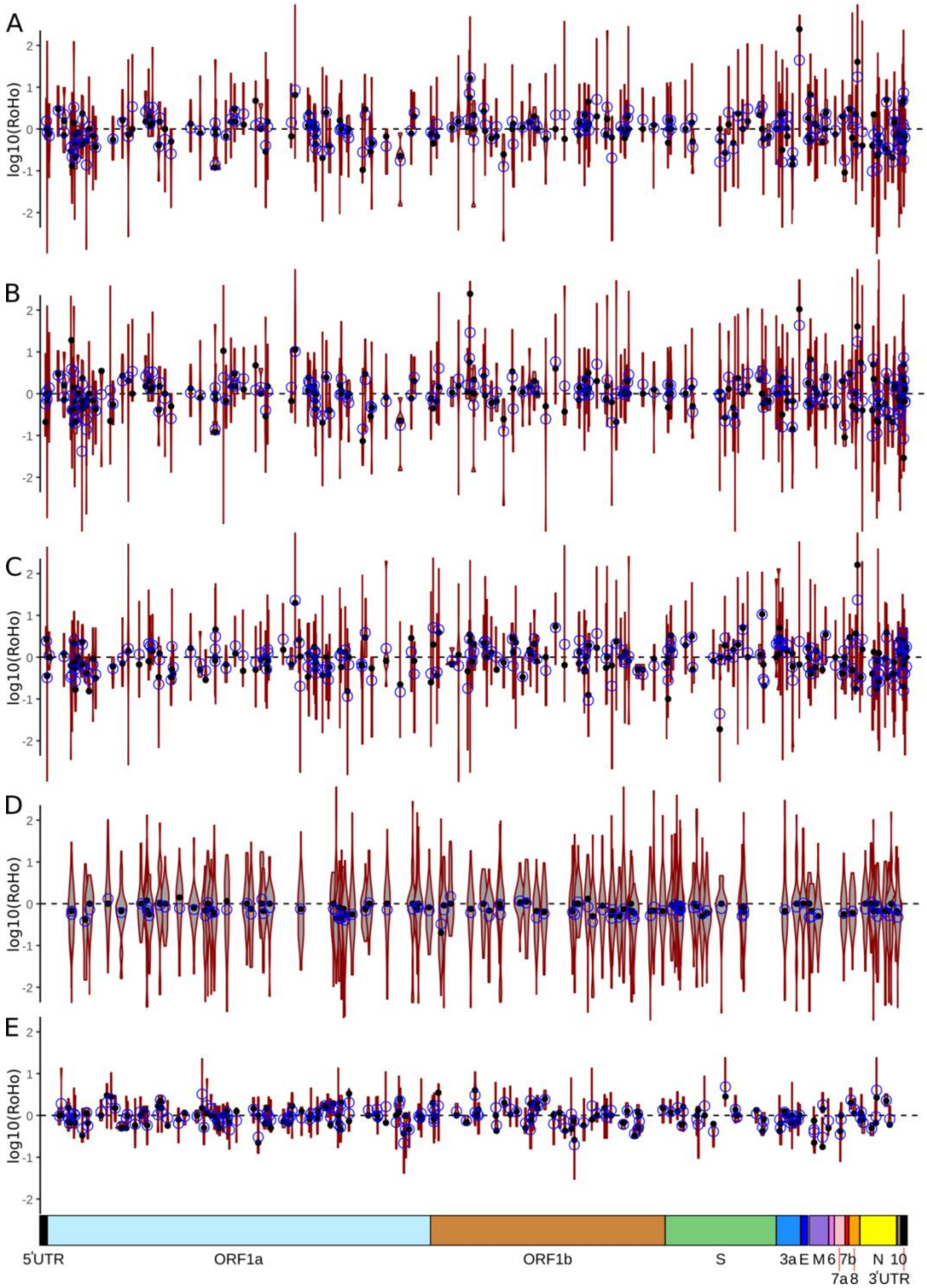

Figure S13. Genome-wide Ratio of Homoplasic Offspring (RoHO) scores for homoplasic mutations for which we only enforced a lower minimal number of three independent emergences in the phylogeny. Unless specified otherwise, clades including a secondary homoplasic emergence for the same allele, or having fewer than two descendant tips of each allele are discarded. Masked (de Maio *et al*.) dataset (A); masked (de Maio *et al.*) dataset without discarding embedded homoplasic emergence (B); NextStrain masked dataset (C); 100 randomly generated discrete traits simulated onto the true maximum likelihood phylogeny (D); simulated 10,000 nucleotide alignment of 500 isolates using a 6E-4 substitution rate, see Methods (E). Black dot: median log10(RoHO) value; blue circle: mean log10(RoHO) value. Bottom coloured boxes correspond to encoded ORFs on Wuhan-Hu-1 reference genome.

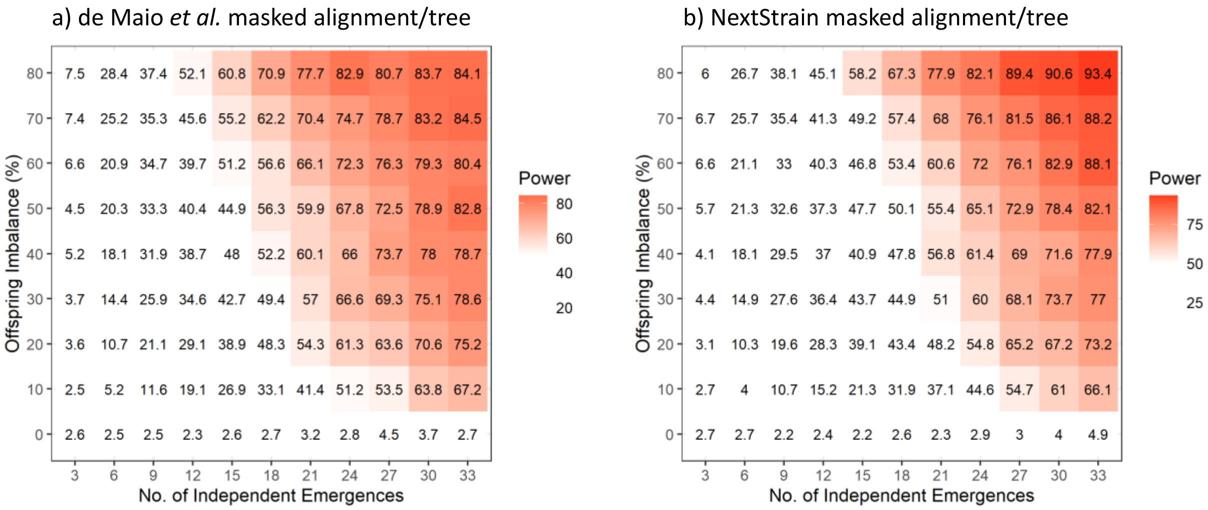

Figure S14. Estimation of the detection power of the paired t-tests applied to the RoHO scores assessed through simulations. Numbers in each box provide the percentage of significant paired t-tests (two-sided, alpha = 0.05) for 1000 replicates for each combination of 3-33 independent homoplasic emergences and an imbalance in the number of offspring carrying either allele between 0-80% (see Methods). a) Provides results for the de Maio *et al*. masking and b) for the NextStrain masking. A list of masked sites is provided in **Data 5**.
