# Supplementary material for: No evidence for increased transmissibility from recurrent mutations in SARS-CoV-2
Source: Nat Commun. 2020 Nov 25;11:5986. doi: 10.1038/s41467-020-19818-2 (PMC7688939; doi:10.1038/s41467-020-19818-2)
Supplement: Supplementary file 3 — Description of Additional Supplementary Files [file 41467_2020_19818_MOESM3_ESM.pdf]

Description of Additional Supplementary Files

File Name: Supplementary Data 1
Description: List of included GISAID accessions together with full metadata and originating and submitting labs. Those accessions excluded from the original download are provided with justifications.

File Name: Supplementary Data 2
Description: Variable positions with synonymous and nonsynonymous annotations across the 46,723 assembly alignment used for the homoplasy detection analysis.

File Name: Supplementary Data 3
Description: Quality metrics of the filtered homoplasies detected in each masked GISAID dataset, see Data 5 for masked positions. Note: positions not annotated as high quality either in the masked or in the alternatively masked GISAID dataset do not appear in the table.

File Name: Supplementary Data 4
Description: Sheet 1: Metrics associated with the Ratio of Homoplasic Offspring (RoHO) scores for 185 homoplasies. These 185 homoplasies were selected from the 398 homoplasies detected in the masked (de Maio et al.) dataset based on phylogenetics parameters (see Methods). Sheet 2: Metrics associated with Ratio of Homoplasic Offspring (RoHO) scores for 199 homoplasies. The 199 homoplasies were selected from the 411 homoplasies detected in the NextStrain masked dataset based on phylogenetics parameters (see Methods).

File Name: Supplementary Data 5
Description: Sites masked from the alignment following the masking strategy suggested by de Maio et al. http://virological.org/t/issues-with-sars-cov-2- sequencing-data/473 (focused on masking putative sequencing errors), time stamped to 30/07/2020 and following the NextStrain masking criteria. Unless otherwise stated, masked suggested by de Maio et al. was used throughout.

File Name: Supplementary Data 6
Description: List of homopolymer regions with coordinates and region length identified in the reference genome Wuhan-Hu-1 (GenBank NC_045512.2, GISAID EPI_ISL_402125).
