# Supplementary material for: No evidence for increased transmissibility from recurrent mutations in SARS-CoV-2
Source: Nat Commun. 2020 Nov 25;11:5986. doi: 10.1038/s41467-020-19818-2 (PMC7688939; doi:10.1038/s41467-020-19818-2)
Supplement: Supplementary file 10 — Reporting Summary [file 41467_2020_19818_MOESM10_ESM.pdf]

nature research

Corresponding author(s): Francois Balloux and Lucy van Dorp

Last updated by author(s): Oct 28, 2020

Reporting Summary

Nature Research wishes to improve the reproducibility of the work that we publish. This form provides structure for consistency and transparency in reporting. For further information on Nature Research policies, see our Editorial Policies and the Editorial Policy Checklist.

Statistics

For all statistical analyses, confirm that the following items are present in the figure legend, table legend, main text, or Methods section.

n/a	Confirmed	
☐	☒	The exact sample size (n) for each experimental group/condition, given as a discrete number and unit of measurement
☐	☒	A statement on whether measurements were taken from distinct samples or whether the same sample was measured repeatedly
☐	☒	The statistical test(s) used AND whether they are one- or two-sided<br>*Only common tests should be described solely by name; describe more complex techniques in the Methods section.*
☒	☐	A description of all covariates tested
☐	☒	A description of any assumptions or corrections, such as tests of normality and adjustment for multiple comparisons
☐	☒	A full description of the statistical parameters including central tendency (e.g. means) or other basic estimates (e.g. regression coefficient) AND variation (e.g. standard deviation) or associated estimates of uncertainty (e.g. confidence intervals)
☐	☒	For null hypothesis testing, the test statistic (e.g. F, t, r) with confidence intervals, effect sizes, degrees of freedom and P value noted<br>*Give P values as exact values whenever suitable.*
☐	☒	For Bayesian analysis, information on the choice of priors and Markov chain Monte Carlo settings
☒	☐	For hierarchical and complex designs, identification of the appropriate level for tests and full reporting of outcomes
☐	☒	Estimates of effect sizes (e.g. Cohen's d, Pearson's r), indicating how they were calculated

Our web collection on statistics for biologists contains articles on many of the points above.

Software and code

Policy information about availability of computer code

Data collection	No explicit software was used for data collection. All genomes were downloaded from GISAID, a genomic data storage repository which provides freely available genomic data on registration and acceptance of the terms of use. We provide the date of download (30/07/2020) in the main text and a full list of accessions in Data 1. This is also detailed in the Data Availability section including a web url link to the GISAID repository.
Data analysis	All bioinformatics software used in this manuscript are fully publicly available. This includes MAFFT v7.471, Augur v6.3.0, IQTree 2.1.0 Covid release, Tomahawk 0.7.0, BactDating 1.01, HomoplasyFinder v0.0.0.9, BioPython v1.7.6, Ape v5.3, Phytools v0.7.2.0 and ggtree v1.16.6. All software are cited with respect to their use in the methods section. In addition we provide all custom scripts used in the analyses as GitHub repositories as stated in the Code Availability statement. All code used to generate the RoHO method presented in this manuscript is available at https://github.com/DamienFr/RoHO, DOI:10.5281/zenodo.4147272. In addition we provide links to the code used to conduct homoplasy filtering (https://github.com/liampshaw/CoV-homoplasy-filtering), per site annotations (https://github.com/cednotsed/nucleotide_to_AA_parser.git) and to assess the number of homopolymer regions in the SARS-CoV-2 genome (https://github.com/cednotsed/genome_homopolymer_counter).

For manuscripts utilizing custom algorithms or software that are central to the research but not yet described in published literature, software must be made available to editors and reviewers. We strongly encourage code deposition in a community repository (e.g. GitHub). See the Nature Research guidelines for submitting code & software for further information.

Data

Policy information about availability of data

All manuscripts must include a data availability statement. This statement should provide the following information, where applicable:

- Accession codes, unique identifiers, or web links for publicly available datasets
- A list of figures that have associated raw data
- A description of any restrictions on data availability

All genomic data analysed is available, on registration, from GISAID (https://www.gisaid.org). A full list of accessions used is provided in Data 1 together with acknowledgement of all originating and submitting laboratories.

Associated raw data to Figure 3 is provided in full in Data 3 and Data 4. Metrics associated to the full alignment are provided as an online and interactive web-resource: https://macman123.shinyapps.io/ugi-scov2-alignment-screen/

Field-specific reporting

Please select the one below that is the best fit for your research. If you are not sure, read the appropriate sections before making your selection.

☒ Life sciences ☐ Behavioural & social sciences ☐ Ecological, evolutionary & environmental sciences

For a reference copy of the document with all sections, see nature.com/documents/nr-reporting-summary-flat.pdf

Life sciences study design

All studies must disclose on these points even when the disclosure is negative.

Sample size	We downloaded all available SARS-CoV-2 genomes available on GISAID marked as 'complete', 'low coverage exclude' and 'high coverage only' on 30/07/2020. This corresponded to a total of 48,454 genomes. The ability of our proposed method to detect significant associations was assessed through simulations and power calculations as documented in the main text methods (section Assessing ROHO Performance). Results are provided in Supplementary Figures S13-S14.
Data exclusions	48,454 SARS-CoV-2 accessions were downloaded from GISAID on 30/07/2020 of which 46,723 passed filtering criteria according to genome length, number of N's and being human rather than animal associated. Criteria are described in full in the methods (section Data Acquisition) which includes removal based on a publicly available list of spurious accessions. This is provided, with link and access date, in the main text methods (https://github.com/nextstrain/ncov/blob/master/config/exclude.txt). The list of full genome accessions used, and those excluded, are provided together with full acknowledgments in Data 1.
Replication	The findings presented here are fully reproducible. The list of genomes analysed are provided in Data 1, all of which are free to download on registration with GISAID. The methods provide a full description of the alignment and phylogenetic tree building procedure followed (section Multiple sequence alignment and maximum likelihood tree) which makes use of openly available software. Site filtering scripts are provided at https://github.com/liampshaw/CoV-homoplasy-filtering as described in the text, as are the ROHO score calculation scripts https://github.com/DamienFr/RoHO. The sites considered and ROHO scores are provided in Data 3 and Data 4. All details are provided in the Data Availability and Code Availability statements. Repetition of experiments was not applicable to this study.
Randomization	Randomisation was not relevant to this study which does not consider control and test populations.
Blinding	The study does not include a group allocation component so blinding was not relevant.

Reporting for specific materials, systems and methods

We require information from authors about some types of materials, experimental systems and methods used in many studies. Here, indicate whether each material, system or method listed is relevant to your study. If you are not sure if a list item applies to your research, read the appropriate section before selecting a response.

Materials & experimental systems

n/a	Involved in the study
☒	☐ Antibodies
☒	☐ Eukaryotic cell lines
☒	☐ Palaeontology and archaeology
☒	☐ Animals and other organisms
☒	☐ Human research participants
☒	☐ Clinical data
☒	☐ Dual use research of concern

Methods

n/a	Involved in the study
☒	☐ ChIP-seq
☒	☐ Flow cytometry
☒	☐ MRI-based neuroimaging
